# Multi-Humanoid Robot Arm Motion Imitation and Collaboration Based on Improved Retargeting

**DOI:** 10.3390/biomimetics10030190

**Published:** 2025-03-19

**Authors:** Xisheng Jiang, Baolei Wu, Simin Li, Yongtong Zhu, Guoxiang Liang, Ye Yuan, Qingdu Li, Jianwei Zhang

**Affiliations:** 1 School of Optoelectronic Information and Computer Engineering, University of Shanghai for Science and Technology, Shanghai 200093, China; 221240064@st.usst.edu.cn (X.J.); 223332475@st.usst.edu.cn (B.W.); yuanye_usst@usst.edu.cn (Y.Y.); 2 Institute of Machine Intelligence, University of Shanghai for Science and Technology, Shanghai 200093, China; 3 Henan Academy of Sciences, Zhengzhou 450046, China; 4 Department of Informatics, University of Hamburg, 20146 Hamburg, Germany

**Keywords:** improved retargeting, motion imitation, multi-person pose estimation, human–robot interaction

## Abstract

Human–robot interaction (HRI) is a key technology in the field of humanoid robotics, and motion imitation is one of the most direct ways to achieve efficient HRI. However, due to significant differences in structure, range of motion, and joint torques between the human body and robots, motion imitation remains a challenging task. Traditional retargeting algorithms, while effective in mapping human motion to robots, typically either ensure similarity in arm configuration (joint space-based) or focus solely on tracking the end-effector position (Cartesian space-based). This creates a conflict between the liveliness and accuracy of robot motion. To address this issue, this paper proposes an improved retargeting algorithm that ensures both the similarity of the robot’s arm configuration to that of the human body and accurate end-effector position tracking. Additionally, a multi-person pose estimation algorithm is introduced, enabling real-time capture of multiple imitators’ movements using a single RGB-D camera. The captured motion data are used as input to the improved retargeting algorithm, enabling multi-robot collaboration tasks. Experimental results demonstrate that the proposed algorithm effectively ensures consistency in arm configuration and precise end-effector position tracking. Furthermore, the collaborative experiments validate the generalizability of the improved retargeting algorithm and the superior real-time performance of the multi-person pose estimation algorithm.

## 1. Introduction

In recent years, increasing attention has been given to utilizing captured human motion data to simplify the complex process of robot motion programming and learning [1,2,3,4]. Motion imitation not only enables robots to learn how to perform tasks by observing human movements but also expands the possibilities for enhancing the responsiveness and autonomy of humanoid robots [1,5,6]. While previous studies have made significant progress in this field [7,8], achieving efficient and precise motion imitation remains a challenging problem [9]. The core challenges in the context of multi-robot simultaneous motion imitation are twofold: (1) Existing retargeting methods struggle to simultaneously achieve geometric configuration similarity and precise end-effector position tracking. (2) Motion imitation relies on pose estimation, and traditional multi-person pose estimation often requires multiple motion capture devices, leading to data synchronization issues and increased system complexity. Moreover, these methods typically lack the generalization capability to accommodate different imitation subjects.

In the field of motion imitation, there are two main approaches: learning-based methods and model-based methods. In the domain of animation, reinforcement learning (RL) has been applied to generate complex human motions and perform various tasks. By using RL to train virtual character controllers, these motions can be replicated while exhibiting distinctive styles [10,11], scalability [12,13,14], and reusability [15,16]. In the context of full-sized humanoid robots, some studies [17,18] have employed imitation learning to transfer human-like styles to controllers. However, precise tracking of human movements remains a significant challenge, primarily due to numerous discrepancies between simulation and the real world. These discrepancies include parameter mismatches and hardware limitations, such as those related to torque and joint constraints. Cheng et al. [19] utilized animation datasets in conjunction with RL-driven techniques to achieve upper-body motion imitation, enabling actions such as handshakes, boxing punches, and dancing. However, their method supports only a single robot, and an increase in the number of imitation targets leads to greater training complexity. Additionally, it cannot track the end-effector position, which limits its ability to perform precise tasks.

Model-based approaches can be subdivided into Cartesian-space-based motion mapping and joint-space-based motion mapping. In Cartesian-space-based motion imitation, the goal for the upper body is to track the end-effector pose of the demonstrator’s hands. J. Koenemann et al. [20] proposed a method based on inverse kinematics (IK) to imitate human hand and foot positions while ensuring stability through a balancing controller. M. Arduengo et al. [21] utilized a quadratic programming (QP) controller for solving inverse kinematics and introduced a variable admittance controller to ensure safety during imitation and interaction.

Their approach was validated through multiple experiments on the TIAGo robot. The advantage of this approach lies in its intuitive task execution. However, it requires obtaining the demonstrator’s end-effector positions and poses, which imposes higher demands on pose estimation. Additionally, it cannot guarantee consistent arm configurations during the imitation process, which makes it challenging to meet obstacle avoidance requirements during motion.

In joint space-based motion imitation, the goal is to track the movement of each joint in the demonstrator’s arms. L. Penco et al. [22] proposed a direct joint angle retargeting method that reduces computational complexity, and they validated it through multiple experiments on the iCub robot. In work by Zhang et al. [23], a novel analytical method was introduced, which geometrically computes the joint angles of a human skeletal model based on the construction of 3D keypoints using link vectors and virtual joints. These angles are then mapped to the humanoid robot Nao. However, since this method was specifically designed for the Nao robot, which lacks an external–internal rotation joint in the shoulder, the algorithm cannot compute the corresponding shoulder rotation angles of the human, making it impossible for the robot to accurately imitate human motions. The advantage of this method lies in its high motion similarity and the ability to maintain consistent arm configurations, which enables obstacle avoidance during the imitation process. However, it cannot ensure accurate tracking of the end-effector position during task execution, making it difficult to perform operational tasks.

One of the key challenges in humanoid robotic arm motion imitation is ensuring both kinematic consistency and precise end-effector tracking. Although previous studies have explored human-to-humanoid retargeting, they have yet to effectively address this issue. To tackle this challenge, we propose an improved retargeting algorithm. Specifically, we first establish a geometric model of the human arm and solve for joint angles using inverse kinematics. These joint angles are then used as the initial conditions for a quadratic programming-based optimization in Cartesian space, ensuring that the final solution satisfies both joint-space constraints and end-effector tracking accuracy.

The advantages of multi-robot collaborative task execution lie in task parallelism and enhanced adaptability, enabling significant improvements in efficiency and fault tolerance [24,25]. In collaborative tasks requiring high precision and adaptability, a key challenge is the real-time correction of robot actions. Recent studies have shown that providing robots with real-time feedback similar to human interaction can improve their performance and safety across various application scenarios [26]. One critical technology in this context is human posture estimation. Conventional multi-person pose estimation typically relies on multiple motion capture devices, which increases system complexity and introduces issues such as data asynchrony [4,27]. Moreover, different imitation targets require generalized mapping algorithms capable of handling diverse motion patterns [28,29].

With regard to posture estimation, a number of methodologies have been proposed in recent times [30,31,32], based on different camera devices, functional principles, and performance. Researchers have obtained new results using a monocular camera [33] or multiple synchronized cameras [34], but the inference time in real-time applications has not been considered in these works. Hwang Y. et al. [35] developed a posture monitoring method that combines information obtained from a monocular camera and the robot, enabling real-time upper limb posture estimation without the need for a training process. In the field of multi-person pose estimation, OpenPose [36] provides real-time 2D multi-person keypoint detection with inference time superior to previous methods, while maintaining high-quality results. However, due to its nature as a heat map-based approach, the inference speed may be reduced, and residual errors may occur. William McNally et al. [37] proposed a new method for multi-person 2D pose estimation that can simultaneously detect keypoint objects and human pose objects. The proposed method performs significantly faster and more accurately on the Microsoft COCO Keypoints benchmark. Although this method achieves good results in 2D human pose estimation, it does not consider extending it to 3D pose estimation.

Another critical challenge in multi-humanoid synchronized imitation is real-time, efficient multi-person 3D pose estimation. To enable humanoid robots not only to perform tasks independently but also to collaborate on complex tasks, we integrate 2D pose estimation with depth information and propose an efficient multi-person 3D pose estimation method. This approach relies solely on a single RGB-D camera to capture and estimate human motion in real time, reducing system complexity while enhancing efficiency.

In summary, we present a comprehensive real-time multi-humanoid robotic arm motion imitation system. By utilizing our improved retargeting algorithm, multiple robots can more accurately and dynamically mimic human arm movements. Additionally, by integrating multi-person pose estimation, the system enables simultaneous imitation and task collaboration among multiple robots. The main contributions of this work include the following:

(1) An improved retargeting algorithm that ensures consistency in the arm’s geometric configuration and precise end-effector tracking in robotic arm motion imitation.
(2) An efficient multi-person 3D pose estimation method, which ensures the system maintains real-time performance and does not significantly increase computational load as the number of detected objects grows.
(3) Experimental validation on a self-developed bipedal humanoid robot platform, demonstrating successful arm motion imitation, task execution, and multi-robot collaboration, thereby verifying the effectiveness and generalizability of the proposed approach.

## 2. Method

### 2.1. Design of Motion Imitation System

**Figure 1:**
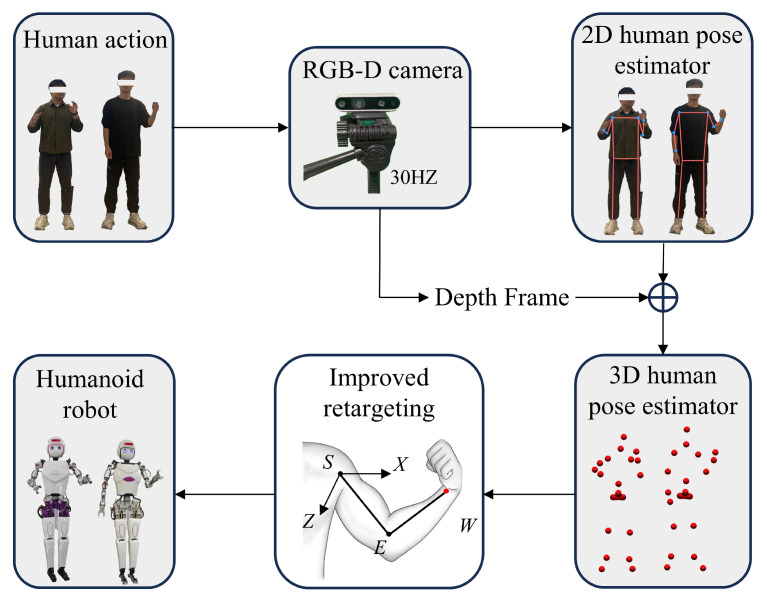
Block diagram of the humanoid robot arm motion imitation system. The motion capture system (RGB-D camera, DFRobot WIKI, Shanghai, China) collects motion data from the demonstrators and transmits it to the upper-level processor. After pose estimation, 3D coordinates of the keypoints are generated and transmitted to the lower-level processor via the ROS protocol. Subsequently, the joint angles of the arm are computed using the improved retargeting algorithm and sent to the robot, enabling it to mimic the arm movements of the demonstrator. It is important to note that, although 3D coordinates are generated for the full-body keypoints, only the arm movements are mapped and reproduced during the motion imitation process.

Figure 1 depicts the robot arm motion imitation system created in this paper. This hardware system is comprised of four distinct parts: the visual capture system, the upper processor, the lower processor, and the mechanical movement system.

The motion capture system can utilize a standard RGB-D camera, and in this paper, the Intel D435 depth camera is used. During the experiment, the camera is positioned directly in front of the subject at a height of approximately 1.5 m above the ground. The captured human body images are transmitted in real time to the upper-level processor via a USB interface.

The upper-level processor is a PC, specifically equipped with an Intel Core i7-12700H processor (2.30 GHz), 16 GB of RAM, and a GeForce RTX 3060 GPU (Intel, Santa Clara, CA, USA). During the experiment, the captured human image data are transmitted to the upper-level processor, where a pose estimation algorithm extracts 2D keypoint data for the human arm joints. Next, the depth information obtained from the camera is fused with the 2D keypoint data to generate 3D keypoints, which are then processed using a Kalman filter. Communication between the upper-level and lower-level processors is achieved through the ROS protocol, with the filtered keypoint data being sent to the lower-level processor.

The lower-level processor is an R86S host equipped with an N6005 CPU. Upon receiving the keypoint data via ROS, the initial joint angles of the arm are calculated based on the geometric model of the human arm, which serves as the initial reference for the end-effector position tracking optimization problem. Through iterative optimization, joint angles that satisfy both the geometric constraints and the end-effector position requirements are obtained. Finally, the target joint angles are transmitted to the mechanical motion system via the CAN bus, driving the motors of the robot arm joints.

**Figure 2:**
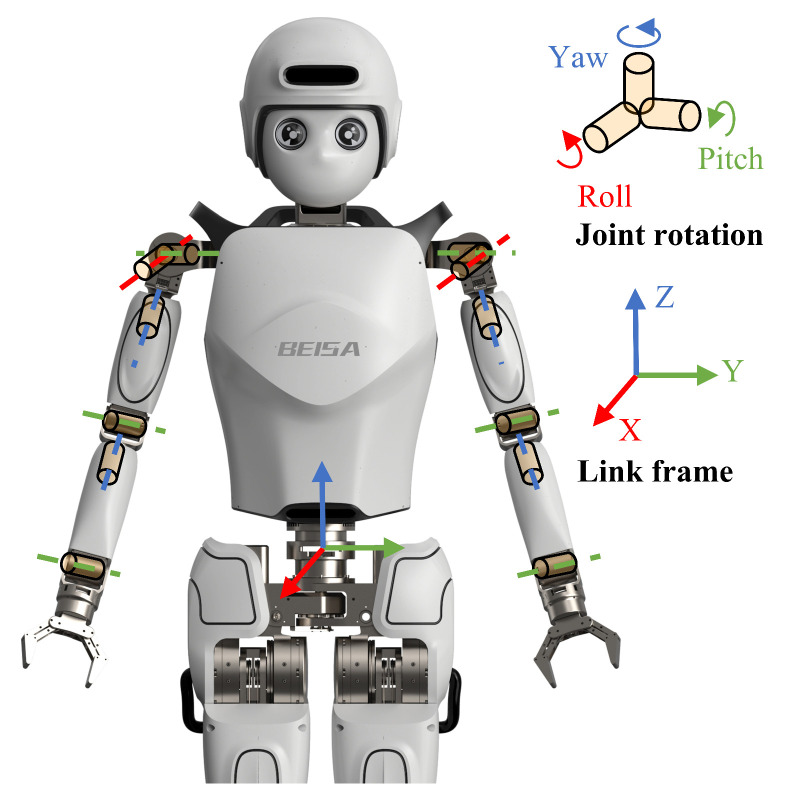
Joint structures and link frames of the robot’s arm.

**Figure 3:**
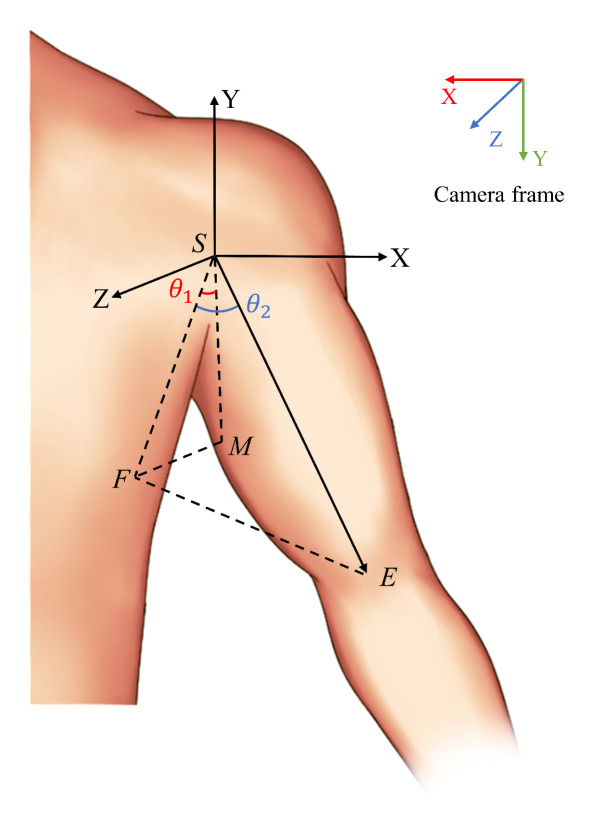
Modeling of the left shoulder joint.

The mechanical motion system comprises the robot’s dual arms, servo motors, and drivers. The humanoid robot employed in this study is a wholly self-designed robot, as illustrated in Figure 2, featuring six degrees of freedom for each mechanical arm and a pair of end effectors. The arm joints include the shoulder flexion–extension joint, shoulder abduction–adduction joint, shoulder external–internal rotation joint, elbow flexion–extension joint, forearm pronation–supination joint, and wrist flexion–extension joint, which together allow for any posture in space (Figure 3).

### 2.2. Multi-Person Pose Estimation

The human pose estimation technique utilizes a fusion of 2D pose estimation and camera depth information to obtain 3D information of human keypoints. The 2D pose estimation is implemented using the Keypoints And Poses As Objects (KAPAO) algorithm. The KAPAO algorithm employs a deep convolutional neural network *N*, with an input consisting of a single RGB image $I\in R^{h\times w\times 3}$ (where *h* and *w* represent the height and width of the image, respectively). The output comprises four types of output grids, denoted as $\hat{G}=\left\{{\hat{G}}^{s}|s\in \left\{8,16,32,64\right\}\right\}$, where ${\hat{G}}^{s}\in R^{\frac{h}{s}\times \frac{w}{s}\times N_{a}\times N_{o}}$ [37]. $N_{a}$ is the number of anchor channels and $N_{o}$ is the number of output channels for each object.

(1) $$\begin{array}{c} N\left(I\right)=\hat{G} \end{array}$$

Each grid utilizes different anchors, with smaller grids exhibiting larger receptive fields optimized for predicting large objects, while larger grids have smaller receptive fields better suited for predicting small objects [38]. Through this set of grids, pose detection objects ${\hat{o}}^{p}$ and keypoint objects ${\hat{o}}^{k}$ are obtained. However, there may be redundancy between ${\hat{o}}^{p}$ and ${\hat{o}}^{k}$, which is addressed by non-maximum suppression to obtain candidate pose objects ${\hat{o}}^{p^{\prime }}$ and keypoint matching objects ${\hat{o}}^{k^{\prime }}$. Finally, a matching algorithm ϕ is used to fuse ${\hat{o}}^{p^{\prime }}$ and ${\hat{o}}^{k^{\prime }}$ into the final pose estimation result.

For the calculation of keypoint coordinates, the output channel ${\hat{g}}_{i,j,a}^{s}$ contains the properties of the predicted object $\hat{O}$, including objectness ${\hat{p}}_{o}$ (the probability that an object exists), intermediate bounding boxes ${\hat{t}}^{\prime }=\left({\hat{t}}_{x}^{\prime },{\hat{t}}_{y}^{\prime },{\hat{t}}_{w}^{\prime },{\hat{t}}_{h}^{\prime }\right)$, object class scores $\hat{c}=\left({\hat{c}}_{1},\ldots ,{\hat{c}}_{K+1}\right)$, and intermediate keypoints for human pose objects ${\hat{v}}^{\prime }={\left\{\left({\hat{v}}_{xk}^{\prime },{\hat{v}}_{yk}^{\prime }\right)\right\}}_{k=1}^{K}$ [37].

Following [39], the intermediate bounding box $\hat{t}$ of an object is predicted in grid coordinates relative to the grid cell origin (i,j) using the following formula:

(2) $$\begin{array}{cccc} {\hat{t}}_{x} & =2\sigma \left({\hat{t}}_{x}^{\prime }\right)-0.5 & {\hat{t}}_{y} & =2\sigma \left({\hat{t}}_{y}^{\prime }\right)-0.5\\ {\hat{t}}_{w} & =\frac{A_{w}}{s}{\left(2\sigma \left({\hat{t}}_{w}^{\prime }\right)\right)}^{2} & {\hat{t}}_{h} & =\frac{A_{h}}{s}{\left(2\sigma \left({\hat{t}}_{h}^{\prime }\right)\right)}^{2} \end{array}$$

$A_{w}$ and $A_{h}$ represent the width and height of the anchor boxes, respectively. The value of *s* is determined based on the corresponding grid, taking values of 8, 16, 32, or 64. The symbol σ denotes the sigmoid function, and ${\hat{t}}_{*}^{\prime }$ represents the intermediate values of the bounding boxes.

This detection strategy is extended to the keypoints of pose objects. The intermediate keypoints $\hat{v}$ for a pose object are predicted in grid coordinates and relative to the grid cell origin (i,j) using the following formula:

(3) $$\begin{array}{cccc} {\hat{v}}_{xk} & =\frac{A_{w}}{s}\left(4\sigma \left({\hat{v}}_{xk}^{\prime }\right)-2\right) & {\hat{v}}_{yk} & =\frac{A_{h}}{s}\left(4\sigma \left({\hat{v}}_{yk}^{\prime }\right)-2\right). \end{array}$$

σ is used to constrain the possible values of the keypoints, and ${\hat{v}}_{*}^{\prime }$ represents the intermediate values of the keypoints.

**Figure 4:**
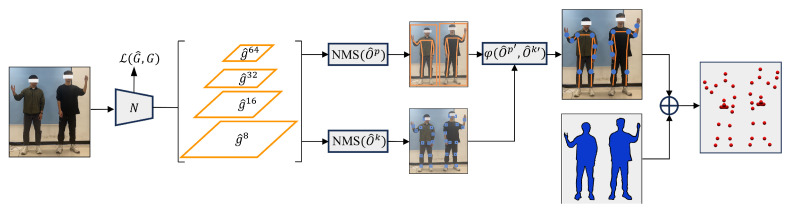
The principle process of multi-person pose estimation. The input image is processed through a YOLO-style feature extractor, resulting in four types of output grids ${\hat{G}}^{s}$, where the superscript *s* takes values from the set {8, 16, 32, 64}. From these grids, the predicted keypoint object ${\hat{o}}^{k}$ and pose object ${\hat{o}}^{p}$ are obtained. Non-maximum suppression is then applied to derive candidate pose objects ${\hat{o}}^{k^{\prime }}$ and keypoint objects ${\hat{o}}^{p^{\prime }}$. A matching algorithm ϕ is employed to fuse ${\hat{o}}^{k^{\prime }}$ and ${\hat{o}}^{p^{\prime }}$ into the final pose result. By incorporating camera depth information, the 3D coordinates of 17 keypoints are obtained. Six keypoints corresponding to the arm are selected as input for the arm kinematics model.

The standard output of KAPAO includes a set of 17 keypoints, which are used to construct a complete human skeleton model. A subset of these 17 keypoints is utilized in the system, corresponding to the 6 keypoints required for the upper body motion imitation on the robot through direct human pose mapping. It should be noted note that Figure 4 illustrates that RGB and depth images are captured simultaneously in the current system. After obtaining the RGB frame, the image is passed to the KAPAO algorithm for processing. The algorithm then outputs a list of 17 2D keypoints, which are overlaid on the original image to outline the recognized human skeleton. Subsequently, using the depth information, these keypoints are transformed in depth space to obtain the 3D coordinates of the human keypoints. The 3D coordinates of three keypoints on the arm (shoulder, elbow, and wrist) are used as input for the improved retargeting method.

### 2.3. Improved Retargeting

#### 2.3.1. Geometric Modeling of Human Arm Motion

Since the shoulder and elbow primarily influence the movement of the arm, while the wrist mainly affects the hand’s motion, this study focuses on the geometric modeling of the shoulder and elbow joints. To clearly illustrate the modeling approach for the shoulder and elbow, the following presents the modeling process for the left arm.

##### Modeling of Human Shoulder Joints

As illustrated in Figure 3, the *S* point represents the left arm shoulder joint, the *E* point is the left arm elbow joint, and the *M* point is a point along the Y axis with a length of *a*.

According to the aforementioned definition, it can be demonstrated that |SM|=a. The coordinates of each point in the camera coordinate system are obtained through pose estimation as follows: $S(x_{0},y_{0},z_{0})$, $E(x_{1},y_{1},z_{1})$, $M(x_{0},y_{0}-a,z_{0})$. The coordinates of each point in the shoulder coordinate system, with the *S* point as the origin, are as follows: the coordinates of point *S* are (0,0,0), those of point *E* are $E(x_{0}-x_{1},y_{0}-y_{1},z_{1}-z_{0})$, and those of point *M* are M(0,−a,0).

The projection of point *E* onto the plane ZSM is point *F*, with the coordinates $F\;(0,y_{0}-y_{1},z_{1}-z_{0})$. The projection of the vector SE onto the plane ZSM forms an angle $\theta_{1}$ with the negative direction of the Y axis, denoted as $∠FSM=\theta_{1}$, where $\theta_{1}$ represents the shoulder flexion–extension joint angle. The angle between SE and its projection onto the plane ZSM is $\theta_{2}$, denoted as $∠FSE=\theta_{2}$, where $\theta_{2}$ represents the shoulder abduction–adduction joint angle.

In the context of geometric relationships, the value of $\theta_{1}$ in ΔFSM can be determined.

(4) $$\begin{array}{cc} \theta_{1} & =\arccos\left[\frac{{\left|\mathrm{SF}\right|}^{2}+{\left|\mathrm{SM}\right|}^{2}-{\left|\mathrm{FM}\right|}^{2}}{2|\mathrm{SF}|\cdot \left|\mathrm{SM}\right|}\right]\\ \; & =\arccos\left[\frac{\left(y_{0}-y_{1}\right)}{\left|\mathrm{SF}\right|}\right] \end{array}$$

The direction of $\theta_{1}$ can be determined by examining $z_{1}-z_{0}$. When $z_{1}-z_{0}>0$, it indicates that the arm is swung forward. Conversely, when $z_{1}-z_{0}<0$, it indicates that the arm is swung backward.

In ΔFSE, settle $\theta_{2}$:

(5) $$\begin{array}{cc} \theta_{2} & =\arccos\left[\frac{{\left|\mathrm{SF}\right|}^{2}+{\left|\mathrm{SE}\right|}^{2}-{\left|\mathrm{FE}\right|}^{2}}{2|\mathrm{SF}|\cdot \left|\mathrm{SE}\right|}\right]\\ \; & =\arccos(\frac{\mathrm{SF}}{\mathrm{SE}}) \end{array}$$

where,

(6) $$\begin{array}{cc} \left|\mathrm{SM}\right| & =\sqrt{a^{2}}=\left|a\right| \end{array}$$

(7) $$\begin{array}{cc} \left|\mathrm{SF}\right| & ={\left[{\left(y_{0}-y_{1}\right)}^{2}+{\left(z_{1}-z_{0}\right)}^{2}\right]}^{1/2} \end{array}$$

(8) $$\begin{array}{cc} \left|\mathrm{FM}\right| & ={\left[{\left(-a-\left(y_{0}-y_{1}\right)\right)}^{2}+{\left(z_{0}-z_{1}\right)}^{2}\right]}^{1/2} \end{array}$$

(9) $$\begin{array}{cc} \left|\mathrm{SE}\right| & ={\left[{\left(x_{0}-x_{1}\right)}^{2}+{\left(y_{0}-y_{1}\right)}^{2}+{\left(z_{1}-z_{0}\right)}^{2}\right]}^{1/2} \end{array}$$

(10) $$\begin{array}{cc} \left|\mathrm{FE}\right| & =\sqrt{{\left(x_{0}-x_{1}\right)}^{2}}=\left|x_{0}-x_{1}\right| \end{array}$$

##### Modeling of the Human Elbow Joint

**Figure 5:**
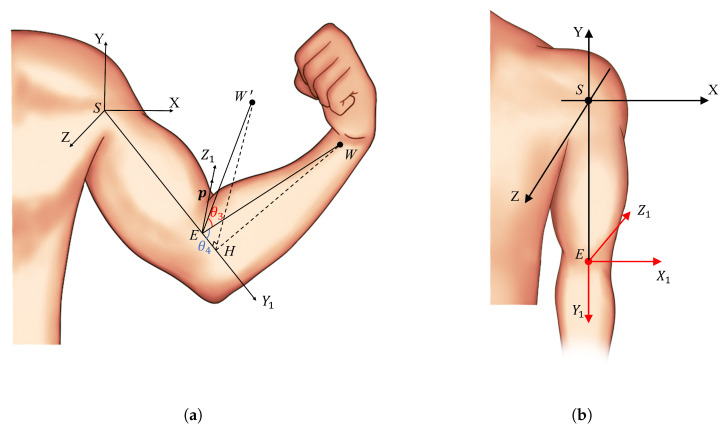
Modeling of elbow Joints. (**a**) Elbow joint coordinate system when the arm joint angles are non-zero, where $\theta_{3}$ represents the shoulder external–internal rotation angle, and $\theta_{4}$ is the complementary angle of the elbow flexion–extension joint. (**b**) Elbow joint coordinate system when all arm joint angles are zero.

As shown in Figure 5a, *W* represents the actual position of the wrist, and $W^{\prime }$ represents the assumed initial position of the wrist (where $\theta_{3}=0$). The angle $\theta_{3}$ is defined as the angle between the vector EW, which points from the elbow to the wrist, and its initial position vector ${\mathrm{EW}}^{\prime }$. The angle $\theta_{4}$ is defined as the angle between vector EW and the extended line of vector SE.

When the wrist is in position $W^{\prime }$, the coordinate system of the elbow joint is denoted as $EX_{1}Y_{1}Z_{1}$. Assuming the initial state of the arm is such that $\theta_{1}$, $\theta_{2}$, $\theta_{3}$, and $\theta_{4}$ are all zero, the elbow joint coordinate system is as shown in Figure 5b.

Using pose estimation, the absolute coordinates of points *S*, *E*, and *W* in the camera coordinate system can be obtained as $S\;(x_{0},y_{0},z_{0})$, $E\;(x_{1},y_{1},z_{1})$, and $W\;(x_{2},y_{2},z_{2})$, respectively. The coordinates of the wrist *W* in the shoulder-relative coordinate system are given by $W\;(x_{0}-x_{2},y_{0}-y_{2},z_{2}-z_{0})$. As shown in Figure 5a, in the shoulder-relative coordinate system, $\theta_{4}$ can be calculated using the dot product of vectors SE and EW

(11) $$\begin{array}{c} \theta_{4}=\arccos\left[\frac{\mathrm{SE}\cdot \mathrm{EW}}{\left|\mathrm{SE}||\mathrm{EW}\right|}\right] \end{array}$$

The range of motion for the human elbow joint is $\pi -\theta_{4}$, where the coordinates of the vector EW are $\left(x_{1}-x_{2},y_{1}-y_{2},z_{2}-z_{1}\right)$.

(12) $$\begin{array}{c} \left|\mathrm{EW}\right|={\left[{\left(x_{1}-x_{2}\right)}^{2}+{\left(y_{1}-y_{2}\right)}^{2}+{\left(z_{2}-z_{1}\right)}^{2}\right]}^{1/2} \end{array}$$

To solve for $\theta_{3}$, given the actual wrist position *W*, we first need to compute HW. As shown in Figure 5a, the $Y_{1}$ axis is defined as the line always pointing from the shoulder joint to the elbow joint, meaning the $Y_{1}$ axis is along the vector SE. Since $\theta_{4}$ is already determined, regardless of changes in $\theta_{3}$, the angles between EW, ${\mathrm{EW}}^{\prime }$, and EH ($Y_{1}$ axis) will remain $\theta_{4}$. In other words, the line ${\mathrm{EW}}^{\prime }$, connecting the elbow and the wrist, rotates around the $Y_{1}$ axis, serving as the rotation axis, to form EW.

(13) $$\begin{array}{c} |{\mathrm{EW}}^{\prime }\left|=|\mathrm{EW}\right| \end{array}$$

(14) $$\begin{array}{c} \left|\mathrm{EH}|=\right|{\mathrm{EW}}^{\prime }|*cos\theta_{4} \end{array}$$

(15) $$\begin{array}{c} \mathrm{EH}=\left|\mathrm{EH}\right|*\frac{\mathrm{SE}}{\left|\mathrm{SE}\right|} \end{array}$$

Thus, by using HW=EW−EH, the vector HW can be obtained.

**Figure 6:**
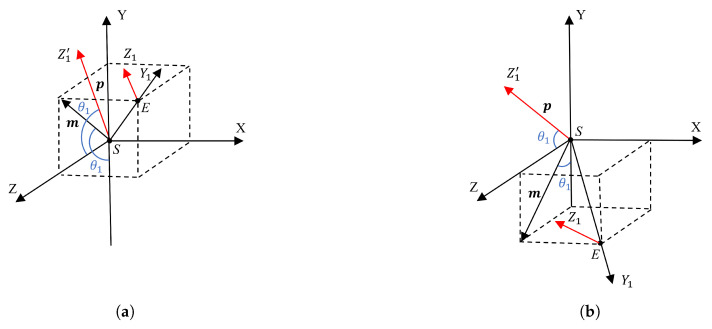
The rotational schematic of the shoulder coordinate system during arm motion. (**a**) Shoulder pitch motion as an acute angle, (**b**) shoulder pitch motion as an obtuse angle.

It is important to note that while the shoulder coordinate system remains fixed during arm movement, the elbow coordinate system changes its orientation, as shown in Figure 6. During arm movement, the elbow coordinate system rotates to a new state. In Figure 6, the direction of the $Z_{1}$ axis changes according to the value of $\theta_{1}$. The $Z_{1}^{\prime }$ axis represents the translation of the $Z_{1}$ axis from the elbow joint to the shoulder joint. The direction vector along the $Z_{1}^{\prime }$ axis is denoted as p, and p⊥m and |p|=1. Therefore, $p=(0,\sin\theta_{1},-\cos\theta_{1})$. As shown in Figure 6, p represents the direction of the $Z_{1}$ axis at the elbow joint when the wrist reaches position $W^{\prime }$. Given HW and the direction vector p, which is parallel to ${\mathrm{HW}}^{\prime }$, $\theta_{3}$ can be determined.

(16) $$\begin{array}{c} \theta_{3}=\arccos\left[\frac{\mathrm{HW}\cdot p}{\left|\mathrm{HW}|\cdot |p\right|}\right] \end{array}$$

#### 2.3.2. End-Effector Cartesian Position Tracking

After obtaining the arm joint angles through human geometric modeling, these angles can be directly used as target angles to control the movement of the robotic arm. While this method can mimic the motion, it does not achieve precise end-effector position tracking, which makes it difficult to perform tasks with high accuracy. To address this issue, we introduce end-effector position tracking constraints.

Considering the differences between the robot and human models, such as size, degrees of freedom, and physical parameters, the end-effector position may differ even if the joint angles are the same. To meet the Cartesian position tracking requirements during task execution, we use the change in the end-effector position (relative to the set initial arm position $P_{human\_init}$) as the control target.

(17) $$\begin{array}{c} P_{robot}=P_{human}-P_{human\_init} \end{array}$$

By monitoring the variation in the human arm’s end-effector position, precise tracking of the robot’s arm movement in task space is achieved. Based on this, the four joint angles obtained from the geometric modeling of the arm are used as initial conditions for the quadratic programming (QP) optimization. This method ensures that the geometric configuration of the robot arm remains consistent with that of the human arm while tracking the Cartesian target position.

The velocity mapping relationship between the joint space and task space can be obtained through differential kinematics.

(18) $$\begin{array}{cc} {\dot{q}}_{r} & =\underset{\dot{q}}{\mathrm{argmin}}{∥J\dot{q}-\dot{v}∥}^{2}\\ s.t. & \\ \; & {\dot{q}}_{min}\leq \dot{q}\leq {\dot{q}}_{max} \end{array}$$

Compute the Lagrangian function of the Equation (18)

(19) $$\begin{array}{cc} L & =\frac{1}{2}{\left(J\dot{q}-v\right)}^{T}\left(J\dot{q}-v\right)\\ \; & =\frac{1}{2}\left({\dot{q}}^{T}J^{T}-v^{T}\right)\left(J\dot{q}-v\right)\\ \; & =\frac{1}{2}\left(\dot{q}J^{T}J\dot{q}-{\dot{q}}^{T}J^{T}v-v^{T}J\dot{q}+v^{T}v\right)\\ \; & =\frac{1}{2}\dot{q}J^{T}J\dot{q}-{\left(J^{T}v\right)}^{T}\dot{q}+\frac{1}{2}v^{T}v \end{array}$$

According to OSQP [40], where the Hessian matrix $H=J^{T}J$ and the gradient vector $g=-J^{T}v$, the bounds are given by the following:

(20) $$\begin{array}{cc} q_{min} & \leq q\leq q_{max}\\ q_{min} & \leq \left(q_{t-1}+▵q\right)\leq q_{max}\\ q_{min}-q_{t-1} & \leq ▵q\leq q_{max}-q_{t-1}\\ \frac{q_{min}-q_{t-1}}{▵t} & \leq \dot{q}\leq \frac{q_{max}-q_{t-1}}{▵t}\\ l & \leq \dot{q}\leq u \end{array}$$

The overall algorithm flow is shown in Algorithm 1, ultimately solving for the arm joint angles *q* that satisfy both the geometric configuration constraints and the end-effector position constraints.
**Algorithm 1** Fusion of geometric modeling and quadratic programming.**Require:** Reference joint angles obtained through geometric modeling $q_{init}=\left\{\theta_{1},\theta_{2},\theta_{3},\theta_{4}\right\}$
**Ensure:**1:Compute the initial position using forward kinematics: $P_{\mathrm{init}}=f\left(q_{init}\right)$2:Set simulation parameters:3:      Step size t=0.05, number of steps step=204:      Previous position $P_{t-1}=P_{\mathrm{init}}$5:Compute position increment: $\Delta P=\frac{\left(P_{\mathrm{robot}}-P_{\mathrm{init}}\right)}{\mathrm{step}}$6:**for** i=1:step **do**7:  **Step 1:** Compute the updated Cartesian velocity and the Hessian matrix:$$v=\frac{\left(P_{\mathrm{init}}+i\cdot \Delta P\right)-P_{\mathrm{pre}}}{t}$$8:  **Step 2:** Compute the joint velocities:$$\dot{q}=\mathrm{osqp}\left(H,g,l,u\right)$$9:  **Step 3:** Update the information:10:     Update joint positions: $q=q+(\dot{q}\cdot t)$11:     Update the current Cartesian position: $P_{t-1}=f\left(q\right)$12:**end for**13:**return** *q*

## 3. Experiments

### 3.1. Single-Person Motion Imitation

In order to validate the accuracy of the human arm kinematic modeling and pose estimation algorithms, we first perform experimental verification through single-person motion imitation. The steps are as follows:

Step 1: Initialize the robot, ensuring all joints move to their zero positions.

Step 2: The demonstrators stand in front of the motion capture system (camera), ensuring that their bodies are within the camera’s field of view, and then activate the camera. The demonstrators begin performing the action, while the upper-level processor uses the pose estimation algorithm described in Section 2.2 to obtain the keypoint coordinates of the shoulder, elbow, and wrist.

Step 3: In the lower-level processing, the keypoint coordinates of the shoulder, elbow, and wrist obtained from pose estimation are used to apply the human arm geometry model described in Section 2.3.1. This allows for the calculation of the shoulder flexion–extension joint angle ($\theta_{1}$), shoulder abduction–adduction joint angle ($\theta_{2}$), elbow flexion–extension joint angle ($\theta_{4}$), and shoulder internal–external rotation joint angle ($\theta_{3}$). Then, through the end-effector position tracking described in Section 2.3.2, the adjusted target joint angles *q* for the robot are obtained.

Step 4: The computed target joint angles of the arm are directly transmitted to the robot, completing the motion imitation process.

**Figure 7:**
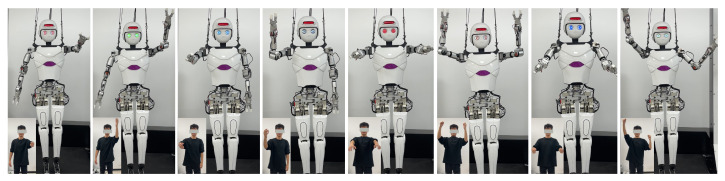
Single-person arm action imitation.

As shown in Figure 7, eight frames were selected from the continuous actions of the demonstrator to illustrate the process of the robot imitating human arm movements. The first and second sets demonstrate the robot imitating the human left arm movement, while the third and fourth sets showcase the robot imitating the human right arm movement. The final four sets present the robot simultaneously imitating the dual-arm actions.

By comparing the arm movements of the demonstrator and the robot, it is evident that the robot can accurately replicate human arm movements through the action imitation method. This validates that the pose estimation algorithm can precisely identify the three-dimensional coordinates of the keypoints of the human arm (shoulder, elbow, and wrist), and the improved redirection algorithm can correctly calculate the arm joint angles, enabling the robot to successfully replicate human arm motions.

### 3.2. Multi-Person Motion Imitation

**Figure 8:**
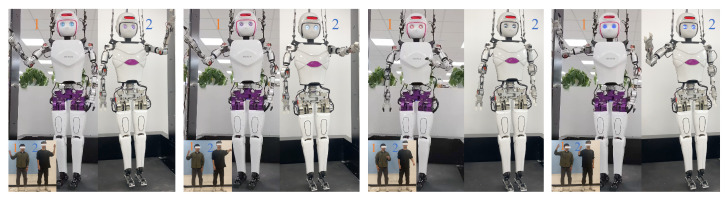
Dual-person arm motion imitation. Robot 1 imitates the actions of demonstrator 1, and Robot 2 imitates the actions of demonstrator 2.

In the dual-person action imitation, although both robots have identical structural dimensions, the two demonstrators differ in height proportions and arm joint sizes. Figure 8 illustrates four sets of different action states, each featuring distinct arm movements. Despite the physical differences between the two demonstrators, both robots can smoothly replicate the arm movements and end-effector positions of the demonstrators. This demonstrates the versatility of the improved redirection method and the real-time performance of multi-person pose estimation using a single camera.

**Figure A1:**
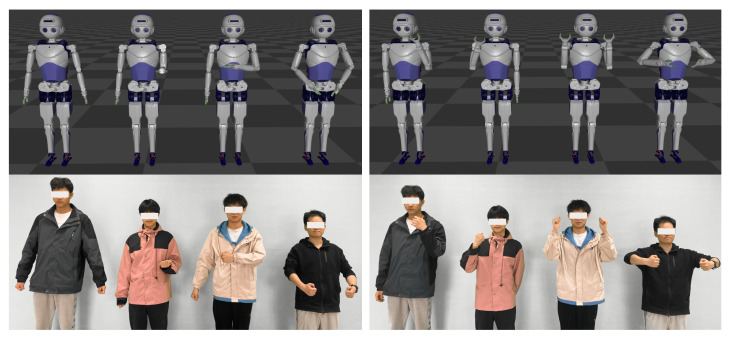
Multiple arm motion imitation simulation. Select the two sets of movements.

To evaluate the real-time performance of pose estimation as the number of detected objects increases, as well as the generalization ability of the action imitation algorithm, we conducted a multi-person action imitation experiment in a simulation environment. As shown in Figure A1, four robot models were created in the Mujoco simulation environment. The movements of four demonstrators were captured by a motion capture system and used as input for pose estimation to generate keypoints for the arms. These keypoints were then processed using the improved redirection method to generate four sets of target arm joint angles, which drive the movement of the simulated robot joints. The joint control system operates with a 500 Hz PD position controller to ensure that its refresh rate matches the position control frequency of the physical robot, ensuring synchronization between the simulation and the actual control.

In the experiments, despite an increase in the number of demonstrators detected, the frame rate of multi-person pose estimation consistently remained at 30 FPS, with no significant impact on real-time performance. Furthermore, the improved redirection algorithm employed effectively ensured the similarity and generalization capability of motion imitation.

### 3.3. Task Operation

To evaluate the feasibility of our multi-person motion imitation system in real-world task scenarios, we selected four tasks: two single-arm imitation tasks (pouring and classification) and two dual-arm collaboration tasks (handover and transport). These tasks cover a range of scenarios that may occur in practical applications. To facilitate task execution, we used a handheld controller to operate the robot’s forearm pronation–supination joint, wrist flexion–extension joint, and the end effector’s gripper.

**Figure 9:**
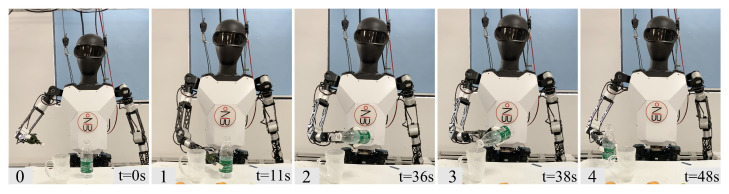
Pouring. (0) The robot initializes in front of the workstation, (1) first grasps the water bottle on the table, (2) moves above the target cup, (3) then rotates the bottle to pour water into the cup, (4) finally releases the bottle. The whole process takes 48 s.

For the pouring task, as shown in Figure 9, the robot needs to pour water from a bottle into a designated cup. This task presents certain risks for the robot and requires stability and accuracy.

**Figure 10:**
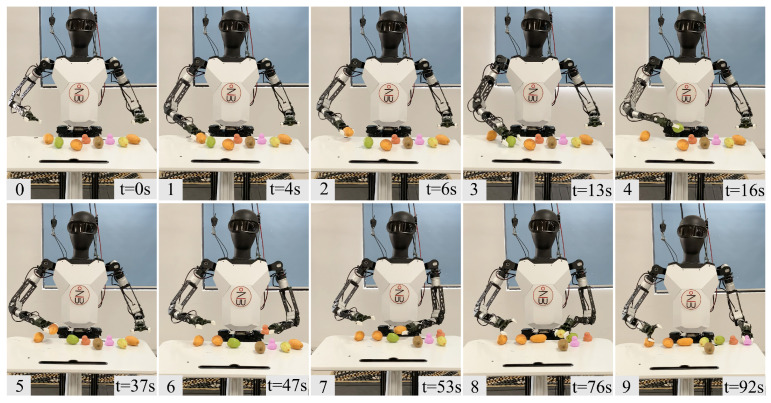
Object classification. (0) The robot initializes in front of the workstation, (1) first selects the object on the right side for sorting, grasps the mango on the far right, (2) then picks it up and places it at the target position. (3) Then it grasps the jujube, (4) picks it up and places it at the target position. The subsequent steps (5–9) repeat the process of (1) (2), sequentially completing the sorting of mangoes (7), jujubes (8), and toys (9). Finally, the sorting of all items is completed, and the entire process takes 92 s.

For the object classification task, also shown in Figure 10, there are four types of items on the table: mangoes, dates, kiwis, and toys. The robot must move these items to their respective areas. This task is similar to everyday classification tasks and requires manipulation with both hands. Due to the restricted workspace of the single-arm robot, even the completion of the task would necessitate a longer time frame.

**Figure 11:**
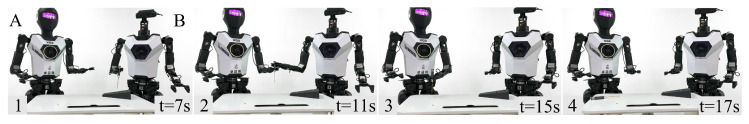
Cooperative handover: Both robots are initialized in front of the Table (1) Robot B picks up the hex screwdriver. (2) Robot A receives the screwdriver passed by Robot B. (3) Robot A prepares to place the screwdriver. (4) Robot A releases its gripper to complete the placement. The entire process takes 17 s.

For the cooperative handover task, as shown in Figure 11, a toolbox is placed on the table containing a hex screwdriver. Robot B needs to pick up the screwdriver from the toolbox and pass it to Robot A. Robot A then places the screwdriver onto the table. This task requires mutual collaboration between the robots.

**Figure 12:**
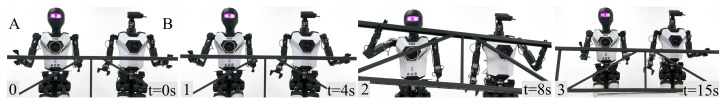
Cooperative transport. (0) Both robots initialize in front of the metal frame. (1) Robots A and B grasp the frame. (2) Both robots lift the frame simultaneously. (3) Both robots place the frame down simultaneously. The entire process takes 15 s.

For the large object transport task, as shown in Figure 12, the table contains a metal frame (length—180 cm, width—40 cm, weighing—3.1 kg). Two robots collaborate to transport this frame. Due to the size and weight of the target object, a single robot would face challenges such as instability in maintaining the center of gravity and insufficient torque in the arm motors. By working together, the two robots can grasp and lift the object cooperatively, reducing the difficulty of the task.

It should be noted that while the appearance of the bipedal robots executing the tasks differs from those previously described, the structural dimensions of the robot arms are identical.

## 4. Results and Discussion

### 4.1. Data Analysis

**Figure 13:**
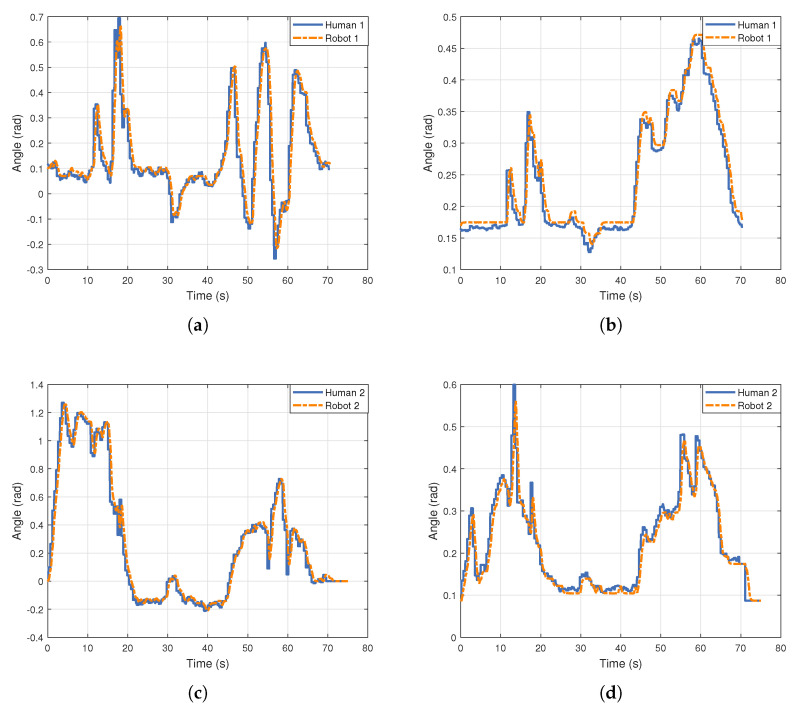
Tracking the trajectories of the left shoulder flexion–extension and abduction–adduction joints in dual-person motion imitation: (**a**,**b**) Robot 1 tracks the joint trajectories of Performer 1’s left shoulder flexion–extension and abduction–adduction joints. (**c**,**d**) Robot 2 tracks the joint trajectories of Performer 2’s left shoulder flexion–extension and abduction–adduction joints.

**Figure A2:**
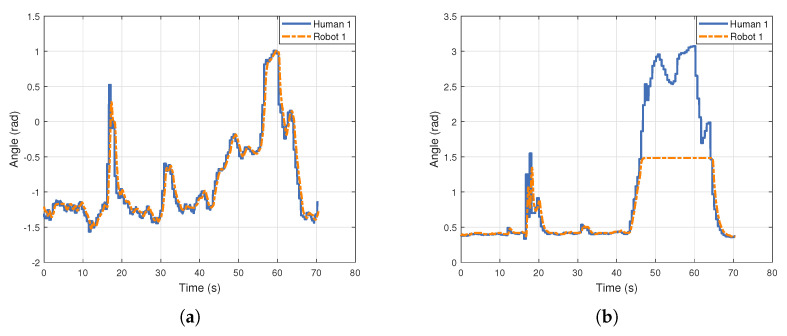

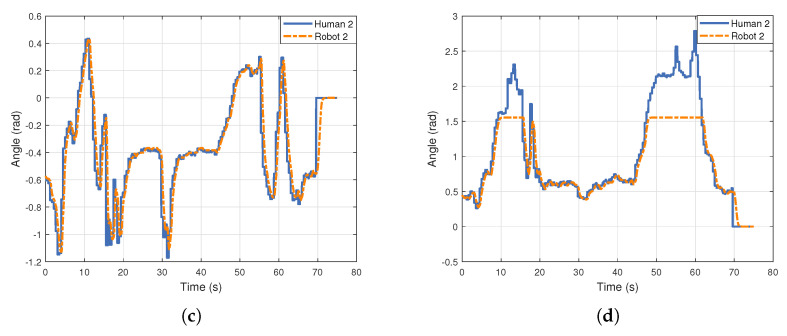
Tracking the trajectories of the left shoulder external rotation–internal rotation and elbow joints in dual-person motion imitation: (**a**,**b**) Robot 1 tracks the joint trajectories of Performer 1’s left shoulder external rotation–internal rotation and elbow joints. (**c**,**d**) Robot 2 tracks the joint trajectories of Performer 2’s left shoulder external rotation–internal rotation and elbow joints.

By examining Figure 13, it is evident that the robot can effectively track the motion trajectories of the human left shoulder flexion–extension and abduction–adduction joints during dual-person motion imitation. The shoulder external rotation–internal rotation joint and the elbow joint also demonstrate good tracking performance, as shown in Figure A2. However, the failure to track the elbow joint in (b) and (d) is due to the joint angle constraints, which prevent the joint from exceeding its mechanical limits.

To quantitatively assess the tracking performance of the motion imitation, this paper employs the mean square error (MSE) as an indicator of joint trajectory tracking accuracy. Smaller MSE values indicate that the robot’s joint movements are closer to the target trajectory.

**Figure 14:**
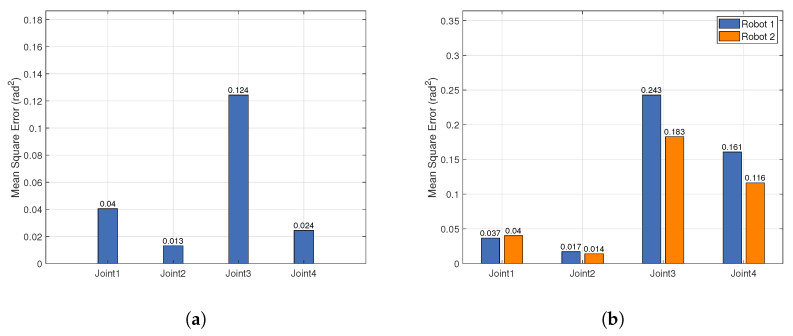
Mean square errors (MSE) of joint angles during arm motion imitation. (**a**) Single-person arm motion imitation. (**b**) Dual-person arm motion imitation. Joint 1 corresponds to the shoulder flexion–extension joint, Joint 2 to the shoulder abduction–adduction joint, Joint 3 to the shoulder external–internal rotation joint, and Joint 4 to the elbow flexion–extension joint.

Figure 14 presents the MSE for each joint during single-person and dual-person motion imitation. Due to the inherent delay in the joint motors, tracking errors are inevitably introduced, which in turn affect the MSE values. The results indicate that, compared to single-person motion imitation, the MSE for each joint during dual-person motion imitation does not increase significantly, thus validating the feasibility and effectiveness of the system in multi-agent imitation. Furthermore, the shoulder abduction–adduction joint has the smallest tracking MSE, while the shoulder external–internal rotation joint has the largest tracking MSE. This is primarily due to the more prominent motion features of shoulder abduction–adduction, which stabilize the human keypoint data output from pose estimation. Consequently, the shoulder abduction–adduction joint reference angles calculated through geometric modeling exhibit smaller fluctuations. In contrast, the larger fluctuations in the wrist depth information result in more pronounced variability in the generated keypoint data, leading to greater fluctuations in the shoulder external–internal rotation joint reference angles. In conclusion, the MSE in joint tracking is mainly attributed to the data fluctuations during pose estimation, with motor response delay also contributing to the tracking accuracy.

**Figure A3:**
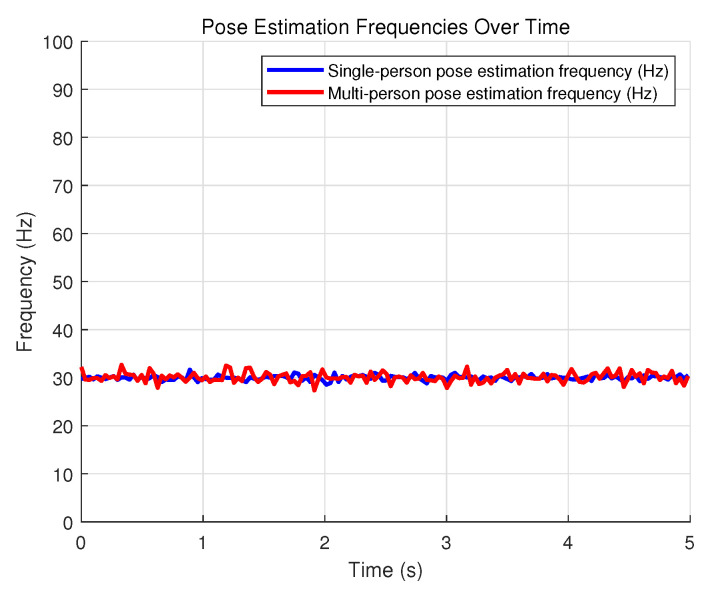
The output frequency of keypoints in pose estimation for single and multiple individuals.

Another critical factor influencing motion imitation performance is the real-time capability of pose estimation. As shown in Figure A3, on a low-power hardware platform equipped with a GeForce RTX 3060 GPU, our pose estimation method maintains a stable keypoint output frequency of approximately 30 Hz in both single-person and multi-person scenarios, as illustrated in Figure 12. Additionally, the low-level motion planning controller operates at an update frequency of 500 Hz, which is crucial for ensuring the robot’s real-time tracking of dynamically changing target points. The combination of real-time pose estimation and high-frequency control updates enables the robot to perform efficient real-time online motion imitation.

### 4.2. Ablation Studies

Through ablation experiments, we compared the performance of using purely geometric methods and optimization-based methods (QP) for end-effector position tracking. As shown in Table 1, the tracking error of the end-effector is $9.35\times 10^{-2}$ when using only the geometric method, whereas the error is significantly reduced to $3.75\times 10^{-4}$ when using the QP optimization method. In contrast, the proposed approach achieves an end-effector tracking error of only $2.065\times 10^{-5}$, which is slightly higher than that of analytical inverse kinematics but significantly outperforms both the geometric and QP methods.

In robotic task execution, precision in end-effector tracking is important, but maintaining a proper arm configuration is equally critical. A well-structured arm configuration not only prevents collisions with the environment and the robot itself but also enhances motion stability and safety. As shown in Table 1, only the proposed improved redirection method can achieve a small end-effector error while preserving a feasible arm configuration.

Furthermore, in terms of computational efficiency, the proposed method requires only about 20 iterations to converge to the optimal solution, making it more suitable for real-time applications compared to traditional QP-based methods.

**Figure 15:**
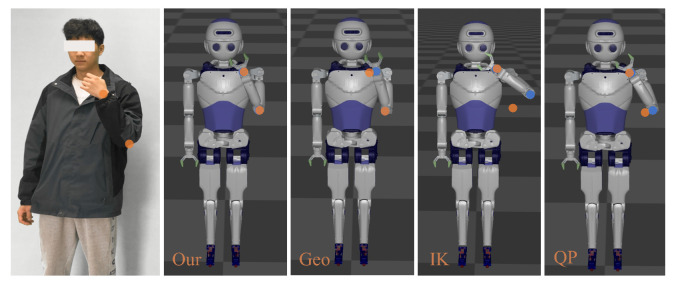
Comparison of different retargeting methods for the same motion keyframes. Orange dots represent target positions, while blue dots represent actual positions.

To provide an intuitive comparison of different retargeting methods in motion imitation tasks, we present an experimental video of our proposed method accessed on 17 March 2025 (Experimental Video (https://youtu.be/lXEJFHylUEI, accessed on 17 March 2025)) and illustrate the differences among various retargeting methods at the same keyframe. As shown in Figure 15, the experimental results demonstrate the following:

- The geometry-based method ensures a similar arm configuration by tracking the shoulder and elbow joint angles. However, since it does not explicitly track the end-effector position, the end-effector tracking error is relatively large.
- The IK method achieves end-effector position tracking; however, it does not incorporate joint limits in the inverse kinematics solution and neglects arm configuration, resulting in significant deviations in the elbow joint position and reduced configuration similarity.
- The QP method successfully tracks the end-effector position while optimizing joint limits and improving arm configuration. However, it increases computational complexity, and the arm configuration still exhibits some errors.
- Our improved retargeting method maintains a low end-effector tracking error while preserving a similar arm configuration, thereby enhancing motion stability and naturalness.

To assess the naturalness and acceptability of multi-robot motion imitation, we invited six participants to perform demonstrations, after which the robots executed the imitation and task operations. The following evaluation criteria were used: (1) Motion smoothness (MS): evaluates whether the robot’s execution exhibits discontinuous or abrupt movements. (2) Pose feasibility (PF): assesses whether the robot maintains an arm configuration similar to that of a human. (3) Task achievement (TA): measures whether the robot accurately completes the designated imitation task. (4) Overall user preference: reflects the participants’ subjective evaluation of the overall imitation quality.

The user rating scale ranges from 1 (poor) to 5 (excellent), with the statistical results presented in Table 2. The experimental results indicate that our method outperforms the geometry-based, IK, and QP methods across all evaluation metrics. In particular, it achieves significantly higher user perception scores in terms of motion smoothness and pose feasibility. These findings suggest that the improved retargeting method not only enhances imitation accuracy but also improves motion naturalness and user acceptability.

### 4.3. Limitation

The proposed improved retargeting algorithm effectively computes the optimal joint angles that simultaneously satisfy both the arm’s geometric configuration and end-effector position tracking. By integrating multi-person pose estimation technology, the framework enables motion imitation and collaborative tasks for multiple robotic arms, enhancing system adaptability and multi-task processing capabilities. Although human motion kinematics has long been a frontier research topic, our study advances retargeting algorithms compared to existing approaches. In particular, by integrating the improved retargeting algorithm with multi-robot collaboration tasks and employing real-time human pose estimation for robotic motion imitation, our method significantly improves motion imitation accuracy and coordination in complex environments.

However, the proposed framework still has certain limitations, particularly in pose orientation tracking. Since our pose estimation system primarily estimates the end-effector position using a single camera, it only captures position data without fully extracting the complete pose information (including rotational components). This limitation notably affects the flexibility of joint motion, especially for complex actions involving forearm pronation–supination and wrist flexion–extension joints, where the robot cannot fully adjust these joints to accommodate task requirements. Future research could incorporate full pose estimation to achieve comprehensive position and orientation tracking, thereby improving the robot’s flexibility and accuracy in complex tasks. Regarding joint limit constraints, as shown in Figure A2b,d, the inability to achieve accurate tracking is primarily due to mechanical constraints restricting joint movement range. This issue is also present in traditional control methods. To address this limitation, future work could introduce more flexible adaptive joint limit control strategies, allowing the robot to adjust its movement range without exceeding its mechanical limits, thereby mitigating the effects of joint constraints.

## 5. Conclusions

This paper presents a comprehensive real-time multi-humanoid robot arm motion imitation framework, which employs an improved retargeting method to successfully achieve an optimal balance between arm kinematic configuration and end-effector accuracy. By integrating multi-person pose estimation technology, the framework not only supports collaborative motion among multiple humanoid robots but also provides an effective solution for multi-robot cooperation in complex tasks.

Experimental results demonstrate that the robots can successfully replicate various human arm movements. Although pose estimation errors increase with the number of demonstrators, the overall motion imitation still maintains high tracking accuracy and end-effector precision. Additionally, in tasks such as pouring water, item classification, and collaboration, we further verified the superior performance of the improved retargeting method in ensuring both arm kinematic structure integrity and end-effector accuracy. Furthermore, with the integration of multi-person pose estimation technology, the system maintains good real-time performance even as the number of detected objects increases, effectively avoiding significant computational load increases.

Although the framework performs excellently in terms of both high accuracy and efficiency, there are still certain limitations in pose tracking. Future research will focus on further optimizing pose estimation techniques to achieve comprehensive tracking of both position and posture, thereby enhancing the robot’s flexibility and precision in complex tasks and enabling it to play a greater role in multi-agent interactions and cooperative tasks.

## Figures and Tables

**Table 1:** Performance comparison of different retargeting methods.

Method	Joint Limits	Configuration	End-Effector Error	Iterations
Geometry	×	✓	$9.35\times 10^{-2}$	×
Analytical IK	×	×	$2\times 10^{-6}$	1
QP	✓	×	$3.75\times 10^{-4}$	≈100
Our	✓	✓	$2.065\times 10^{-5}$	≈20

**Table 2:** User evaluations of different retargeting methods.

Method	MS	PF	TA	Overall
Geo	4	5	2	4
IK	4	2	3	2
QP	3	3	3	3
Our	5	5	4	5

## Data Availability

The original contributions presented in this study are included in the article. Further inquiries can be directed to the corresponding author.
